# Amelioration of Huntington’s disease phenotype in astrocytes derived from iPSC-derived neural progenitor cells of Huntington’s disease monkeys

**DOI:** 10.1371/journal.pone.0214156

**Published:** 2019-03-21

**Authors:** In Ki Cho, Bo Yang, Craig Forest, Lu Qian, Anthony W. S. Chan

**Affiliations:** 1 Department of Human Genetics, Emory University School of Medicine, Atlanta, Georgia, United States of America; 2 Division of Neuropharmacology and Neurologic Diseases, Yerkes National Primate Research Center, Emory University, Atlanta, Georgia, United States of America; 3 Neuroscience Core, Parker H. Petit Institute for Bioengineering & Bioscience, Georgia Institute of Technology, Atlanta, Georgia, United States of America; Texas Technical University Health Sciences Center, UNITED STATES

## Abstract

Huntington’s disease (HD) is a devastating monogenic, dominant, hereditary, neurodegenerative disease. HD is caused by the expansion of CAG repeats in exon 1 of the huntingtin (*HTT*) gene, IT15, resulting in an expanded polyglutamine (polyQ) residue in the N-terminus of the HTT protein. HD is characterized by the accumulation of mutant HTT (mHTT) in neural and somatic cells. Progressive brain atrophy occurs initially in the striatum and extends to different brain regions with progressive decline in cognitive, behavioral and motor functions. Astrocytes are the most abundant cell type in the brain and play an essential role in neural development and maintaining homeostasis in the central nervous system (CNS). There is increasing evidence supporting the involvement of astrocytes in the development of neurodegenerative diseases such as Parkinson’s disease (PD), Huntington’s disease (HD), Alzheimer’s disease (AD), and amyotrophic lateral sclerosis (ALS). We have generated neural progenitor cells (NPCs) from induced pluripotent stem cells (iPSCs) of transgenic HD monkeys as a model for studying HD pathogenesis. We have reported that NPCs can be differentiated *in vitro* into mature neural cells, such as neurons and glial cells, and are an excellent tool to study the pathogenesis of HD. To better understand the role of astrocytes in HD pathogenesis and discover new therapies to treat HD, we have developed an astrocyte differentiation protocol and evaluated the efficacy of RNAi to ameliorate HD phenotypes in astrocytes. The resultant astrocytes expressed canonical astrocyte-specific markers examined by immunostaining and real-time PCR. Flow cytometry (FACS) analysis showed that the majority of the differentiated NPCs (95.7%) were positive for an astrocyte specific marker, glial fibrillary acidic protein (GFAP). Functionalities of astrocytes were evaluated by glutamate uptake assay and electrophysiology. Expression of *mHTT* in differentiated astrocytes induced cytosolic mHTT aggregates and nuclear inclusions, suppressed the expression of *SOD2* and *PGC1*, reduced ability to uptake glutamate, decreased 4-aminopyridine (4-AP) response, and shifted I/V plot measured by electrophysiology, which are consistent with previous reports on HD astrocytes and patient brain samples. However, expression of small-hairpin RNA against *HTT* (shHD) ameliorated and reversed aforementioned HD phenotypes in astrocytes. This represents a demonstration of a novel non-human primate (NHP) astrocyte model for studying HD pathogenesis and a platform for discovering novel HD treatments.

## Introduction

Huntington’s disease (HD) is a devastating monogenic, hereditary, neurodegenerative disease characterized by progressive brain atrophy in striatum, cortex and other brain areas [1]. The psychophysiological phenotypes include cognitive, behavioral, and motor function deficits and psychiatric abnormalities [2,3]. HD affects about 3–10 people in every 100,000 people in Western Europe and North America, and juvenile cases account for 4.92% of cases, with an early age of onset at 20 [4,5]. The juvenile form of HD is associated with more severe chorea, dystonia, and neurodegeneration in the frontal and temporal lobes [5]. The primary etiology of HD is the neurodegeneration of basal ganglia, which partially explains the pronounced motor and cognitive symptoms observed in HD patients [6]. Following the onset of the disease, the atrophy spreads to other cerebral areas, exacerbating HD symptoms.

HD is caused by a CAG expansion in exon 1 of the huntingtin (HTT) gene, IT15, which results in expanded polyglutamine (polyQ) residue in the N-terminus of the HTT protein [2]. The onset and severity of the disease are governed by the size of the trinucleotide repeat. A CAG repeats of 35 or more is expected to develop HD [7]. The typical age of onset for HD is between 35–55 years with the repeat size of 40, while juvenile HD is expected with more than 60 CAG repeats[5]. The accumulation of oligomeric mutant HTT (mHTT) and the formation of nuclear inclusions are hallmarks of the disease [2]. However, the role of mHTT in HD pathogenesis remains unclear. HTT protein has multiple proteolytic cleavage sites or splicing sites, which allows the production of a variety of N-terminal fragments [2]. However, the mHTT creates aberrant splicing and results in the formation of small oligomeric fragments that form aggregates and accumulate in cells and disrupt cellular processes [2]. Studies have reported role of HTT in inhibition of neural hyperexcitation [8], defected ubiquitin-proteasome system in HD mouse model [9], mitochondrial dysfunction in HD patients and animal models [10], disruption of autophagic pathway in HD brain [11], and calcium homeostasis dysfunction in HD mouse [12].

Astrocytes play important roles in the CNS, such as neural development, synapse formation, glutamate removal, neuron supports, brain tissue repairs, and maintaining homeostasis [13]. Increasing evidence suggested damaged glial cells can accelerate atrophy in neurodegenerative diseases such as Alzheimer’s and HD [14]. Recent studies have shown astrocyte dysfunction in HD [15] and mHTT led to the loss of neuron protection against *N*-methyl-D-aspartate (NMDA) toxicity, reduced capacity to buffer extracellular K^+^ [16], impaired glutamate transport, dysfunction in proinflammatory mediators and anti-inflammatory cytokines, astrocytic mitochondrial dysfunction, compromised the release of trophic factors brain-derived neurotrophic factor (BDNF) and chemokine Ccl5/RANTES [13]. Moreover, overexpressing mHTT in astrocytes recapitulate age-dependent neurological symptoms [17], which suggests the importance of astrocytes in HD pathogenesis. A recent study also suggested that differentiating astrocytes from neural stem (NS) cells is a powerful tool to investigate cholesterol biosynthesis dysfunction in HD astrocytes [18]. Therefore, there is increase interest to better understand how astrocytes are involved in HD development and progression.

Recent advancement in cellular reprogramming technology provides a unique opportunity to derive induced pluripotent stem cells (iPSCs) from patient-specific cells. Using directed differentiation methods, the resulted neural cells develop disease phenotypes and have been widely used for studying HD pathogenesis and the development of novel therapeutics [19,20,21,22,23,24]. Neural progenitor cells (NPCs) are multipotent cells found in the subventricular zone (SVZ) in the CNS which are self-renewal and capable to differentiate into neural cells including neurons and glial cells [25]. Our team has derived NPCs from HD monkey iPSCs [19,22,26]. These NPCs were capable of differentiating into multiple neuronal cell types including GABA^+^, DCX^+^, NeuN^+^, DARPP32^+^, and GFAP^+^ neural cells *in vitro* and *in vivo* [19].

Here we report the differentiation of monkey NPCs into functional astrocytes and the amelioration of HD cellular phenotypes using small-hairpin RNA (shRNA). Astrocytes derived from NPCs showed high homogeneity in canonical astrocyte-specific markers GFAP and up-regulation of astrocyte-specific transcripts such as *GFAP*, *S100B*, *APOE*, and *LCN2*. Expression of mature astrocyte-specific markers such as *GLT1*, *GRIA1* and *GRM1* further suggest the functionality of the resulted astrocytes while lower expression level of these mature astrocyte markers in HD astrocytes suggest astrocytic dysfunction in glutamate uptake. Functionality of the differentiated astrocytes is further supported by glutamate update and electrophysiology signatures of astrocytes. Compared to WT-NPC derived astrocytes, HD astrocytes show reduced glutamate uptake capability while overexpressing shHD in HD astrocytes suppressed mHTT aggregates, restored sensitivity to 4-aminopyridine (4-AP) treatment in electrophysiology recording, and increased glutamate uptake ability.

## Materials and methods

### Maintenance of NPC

NPCs were derived from pluripotent stem cells generated from transgenic monkeys[22]. Transgenic monkeys were generated by injecting high titer lentiviruses expressing exon 1 of human HTT with 84 CAG repeats followed by intracytoplasmic sperm injection fertilization[26]. Repeat size of the resulted ES cell line had 77 CAG repeats while WT had 16 CAG repeats. Neural progenitor cells (NPCs) were maintained and expanded as previously described[19]. In brief, cells were cultured on P/L-coated [1 μg/cm^2^ laminin (Sigma) and 20 μg/mL poly-L-ornithine (Sigma)] cell culture dishes with neural proliferation medium [Neurobasal-A medium (Life Technologies) with 1 x penicillin/streptomycin (Invitrogen) and 1 x B27 (Life Technologies), 2 mM of L-glutamine, 20 ug/mL of bFGF (R&D), and 10 ng/mL of mLIF (Chemicon)]. Media was replenished every two days, and depending on the confluence (90–95%), cells were passed 1:1.5 ratio.

### *In vitro* astrocyte differentiation

Astrocyte differentiation protocol was based on previously published protocol[27]. In short, to initiate the differentiation process, NPCs were seeded with the seeding density of 2X10^5^ cells/cm^2^. The differentiation was initiated on P/L-coated tissue culture dishes containing 500 nM of azacytidine (Aza-C) (Sigma), 20 nM of trichostatin (TSA) (Sigma), 20 ng/mL of bone morphogenetic protein 2 (BMP2) (R&D), and 1X of B27 (Life Technologies) in Neurobasal-A medium (Life Technologies) for 2 days. Then, Aza-C and TSA were removed, and cells were cultured with the astrocyte differentiation media for 28 additional days.

### Immunocytochemistry

Cells were fixed with 4% paraformaldehyde / 1x PBS. Fixed cells were incubated at 4°C overnight with primary antibody (in 3% BSA/ 1x PBS) with appropriate dilutions: OCT4 (1:500, Santa Cruz), SOX2 (1:500; Stem Cell Technologies), PAX6 (1:300; Covance), Musashi-1 (1:250, Chemicon), Nestin (1:1000, Milipore), FOXO4 (1:500, Cell Signal), β-III Tubulin (1:300, Milipore), MAP2 (1:500, Milipore), GFAP (1:1000, Chemicon and Sigma), ALDH1L1 (1:500, Origene), S100β (1:100, Abcam), and mEM48 (1:50). The secondary antibody was diluted in the blocking buffer according to its best working concentration: Alexa 488 (1:1000; Life Technologies), Alexa 594 (1:1000, Life Technologies), and Cy5 (1:1000, Sigma). The nucleus was visualized with Hoechst 33342 (5 mg/mL). Samples were examined under an epifluorescent scope (Olympus BX51).

### Fluorescence-activated cell sorting (FACS) analysis

After 30-day differentiation, cells were dissociated with 1x Accutase (Life Technologies) and fixed with 1x BD FACS Permeabilizing Solution (BD Biosciences). Cells were incubated with primary antibody (GFAP) for one hr in 0.5% BSA/PBS. Cells were incubated with secondary antibody for one hr. Cell counts were made with FACSCalibur Flow Cytometer (BD Biosciences). Background fluorescence was subtracted using unlabeled cells and channel compensation was performed using fluorochrome-labeled compensation beads (BD Biosciences). A total of 10,000 events were recorded. Quantifications and data analysis were done on FlowJo Analysis Software (TreeStar).

### RT-qPCR

Total RNA was extracted using TRIzol (Life Technologies) followed by DNA digestion using DNA-free^™^ kit (Invitrogen). cDNA was synthesized using High-Capacity cDNA Reverse Transcription Kits (Applied Biosystems) using 500 ng of RNA samples. RT-qPCR was performed using either IQ^™^ SYBR Green Supermix or TaqMan Gene Expression Master Mix (Applied Biosystems) depending on the primers used. CFX96 Real-Time Detection System (Bio-Rad) was used for the reaction. SYBR Green primer sequences are provided in Table 1, and the Taqman assay information is provided in Table 2.

**Table 1:** SYBR Green primer sequences.

Gene Symbol	Forward	Reverse
*TUBB3*	GCCAAGTTCTGGGAAGTCAT	GGCACGTACTTGTGAGAGGA
HTT Exon 1	GCGACCCTGGAAAAGCTGAT	CTGCTGCTGCTGGAAGGACT
HTT Exon 26	ACCCTGCTCTCGTCAGCTTGG	AGCAAGTTTCCGGCCAAAAT
*UBC*	CCACTCTGCACTTGGTCCTG	CCAGTTGGGAATGCAACAACTTTA
*TH*	GAACTTCTGGGGTCGCTCC	ACCTCAAGACTTACCGGCTT
*NES*	TGGCAAGAGGCCGGTACA	CCGTATTTGTCCTTCACCTTC
*SOD2*	GATCCACTGCAAGGAACAACAG	CAGGCCTGACATTTTTATACTGAAGGT
*GRM1*	CTCGGGCATGCATTGTGAAA	GCGTTCTTGTTAGCAGTCCC
*PPARGC1A*	GCTGAGCTTTCGAGGGAGTT	ACTGTGGGTCCTTAAGGGGG
*FOXO4*	ACCATGGATGTGTTAGGGGC	CCCTGTGTGTAAATGGGGGA
*MAP2*	ATCTTTCTCCTCTGGCTTCCG	GGTGTGGTGGCTGGAAGGTA
*MSI1*	CACAGCCCAAGATGGTGACT	TCCACCTTCCCAAACTGCTC
*LCN2*	AGGGAATGCAGTTGGCAGAA	GGAGGTCACGTTGTAGCTCT
*GFAP*	CCAGCTCGCGGTTCTCATAC	CTCATGGACTTTCAGGGCGT
*S100β*	GGAAGAGGATGTCTGAGCTGGA	CAGCTTGTGCTTGTCTCCCT
*APOE*	GGGTCGCTTTTGGGATTACC	CTCATCCATCAGCGTCGTCA
*GLT1*	ATGCACGACAGTCACCTCAG	AGGATGACACCAAACACCGT
*SOX2*	CACAGCGCCCGCATGTACAA	AGTTCGCTGTCCTGCCCTCA
*HSPD1*	ATTGCCAATGCTCACCGTAAGC	TTGACTGCCACAACCTGAAGAC
*CASP3*	TCGCTTTGTGCCATGCTGAAA	TGTTGCCACCTTTCGGTTAAC
*CASP9*	CAATCCTCTCGACCGACACA	CGACCTGACTGCCAAGGAAA
*BCL2L1*	CGGGATGGGGTAAACTGG	AGGTGGTCATTCAGGTAAGTGG
*DDIT*	TGAACGGCTCAAGCAGGAAATC	TTCACCATTCGGTCGATCAGAGC

**Table 2:** TaqMan assay information.

Gene Symbol	Assay ID
SOD2	AIKAJ6N
PPARGC1A	Rh01016720_m1
FZD1	Rh02914343_s1
GFAP	Rh02840887_m1
GRIA2	Rh02829821_m1
GRM5	Hs00925572_m1
S100β	Rh02799138_m1
SERPINA3	Rh02793826_m1
UBC	Hs01871556_s1

TaqMan Assay IDs used in this study

### Glutamate uptake assay

Glutamate clearance capacity was measured using Glutamine and Glutamate Determination Kit (Sigma) following manufacturer’s protocol[28]. In brief, the culture medium was replaced with 1.5 mL of serum-free HBSS containing 100 mM glutamate (Abcam). Cells were returned to the incubator for two hours at 37 °C, 12.5 μL of supernatant was transferred to 96-well plate, and remaining glutamate in the medium was determined. The absorbance at 340 nM was obtained using BioTek Synergy HT Microplate Reader (BioTek, Winooski, VT). The concentration in the supernatant was calculated using the standard curve, and change in glutamate concentration was calculated. As a control, 293FT cells were treated the same way as the astrocytes. The concentration of total cell protein was determined using BCA Protein Assay Kit and used as a reference (Pierce, Rockland, IL).

### Electrophysiology

Differentiated cells were plated on 4-well plates with cover glasses coated with P/L for 2 days before the electrophysiological recording. Membrane currents were recorded using automated whole-cell patch clamping technique ^77–79^. Patch pipettes were pulled from thin-walled borosilicate glass capillaries (Harvard Apparatus, MI) and fire polished to have a tip resistance between 6~9 MΩ when filled with standard internal solution containing (in mM): 144 KCl, 2 MgCl2, 5 EGTA, 10 HEPES (pH 7.2) with KOH and osmolality of 310 mOsm. Data were sampled at 10 kHz and low pass filtered at 2 kHz for analysis. Series resistance was periodically monitored and if it exceeds 20 MΩ, the recording will be discarded. All the experiments were performed under room temperature (20~24 °C).

Protocols of voltage stimulations include both ramp stimulation (from the holding potential of -60 mV, stepped the membrane potential to -100 mV for 200 ms before a 100 ms long depolarizing ramp to +100 mV) and voltage step stimulation (a voltage-step family with 200 ms steps from -100mV to 120 mV in 20 mV increments from holding potential of -60 mV. The interval between each pulse in both protocols was set at 10 s. The resting membrane potential was measured 2 min after establishing the whole-cell configuration using Multipatch software (Molecular Devices). 4-AP was dissolved in distilled water and applied at the concentration of 500 μM. Percentage of ramp current decrease was measured at +100 mV by application of 4-AP (500 μM) in the superfusion medium. TBOA was dissolved in DMSO and administrated at a concentration of 200 μM in the supervision artificial cerebrospinal fluid (ACSF) medium. Amplitudes of peak ramp currents were used to study TBOA’s effects. Both drugs were purchased from Tocris Bioscience.

### Statistical analysis

Unless otherwise mentioned, one-way analysis of variance (ANOVA) was used for statistical comparison. For paired data, two-tailed Student’s *t*-test was used for statistical comparison. Bar graphs illustrate as a mean ± standard error of the mean (SEM) values. Statistical tests were performed using either GraphPad Prism 6 (GraphPad Software, Inc.) or SPSS 24 (IBM). Statistical significance was established *P* < 0.05.

## Results

### Monkey NPCs derived astrocytes *in vitro*

**Fig 1:**
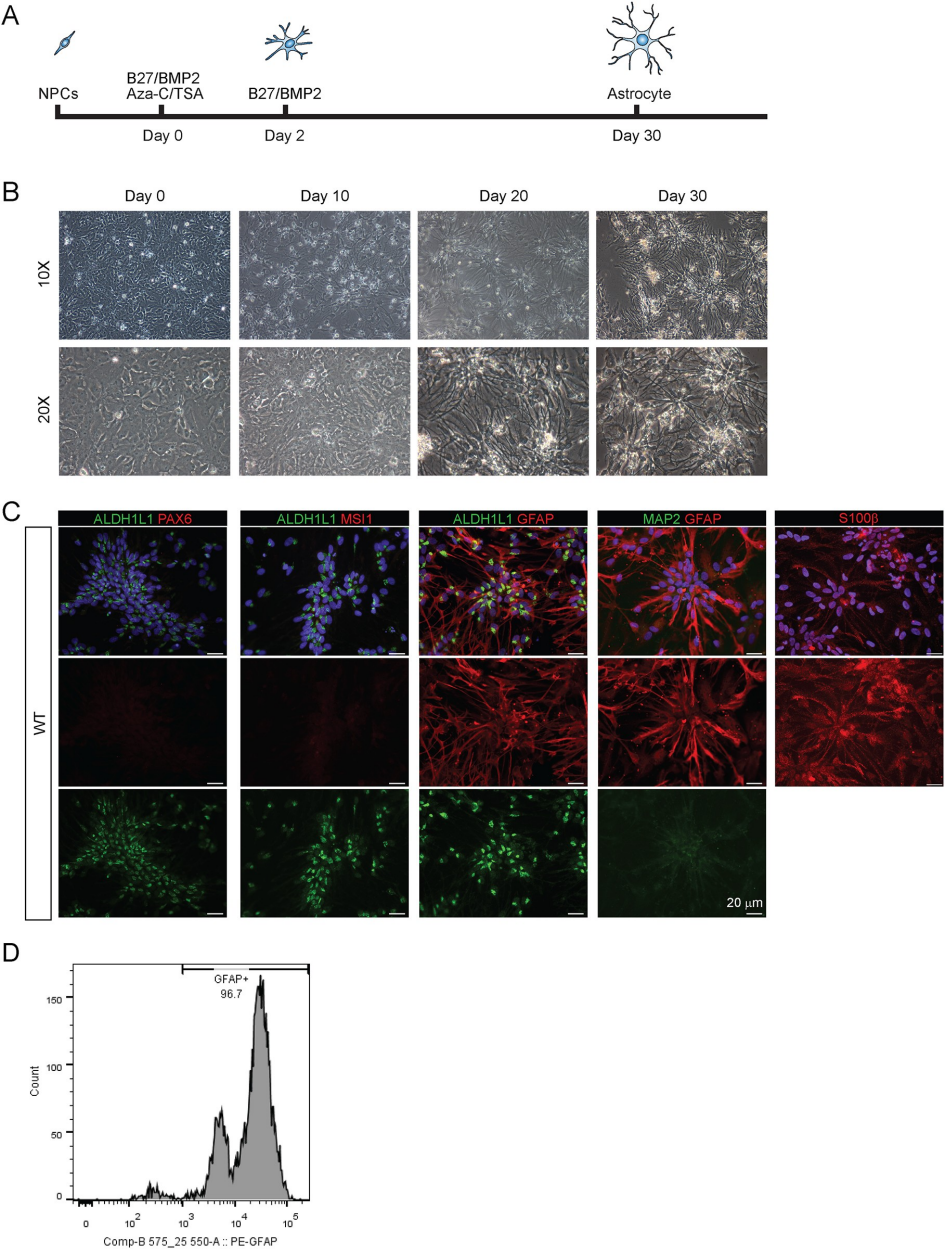
Differentiation of NHP NPCs to astrocytes *in vitro*. (A) Graphical description of differentiation protocol. NPCs were treated with BMP2, bFGF, Aza-C, and TSA for two days. After initial treatment, Aza-C and TSA were removed, and only BMP2 and bFGF were supplemented in the media. (B) Morphological changes during differentiation process showing development and elongation of processes. (C) Wild-type astrocytes are expressing astrocyte-specific proteins GFAP (red), ALDH1L1 (green) and S100β (red), while no detectable NPC (PAX6 and MSI1) (red) and neuronal (MAP2) (green) specific proteins were expressed. (D) Fluorescence-Activated Cell Sorting showing the population of cells that positive for GFAP.

**Fig 2:**
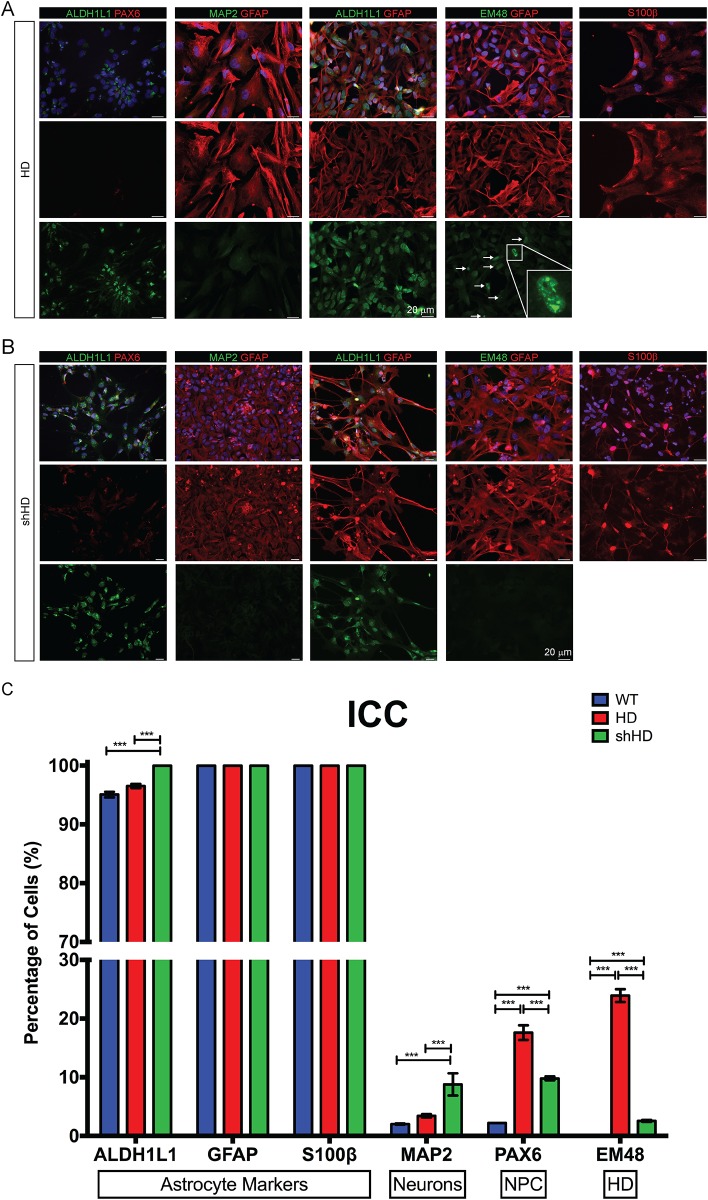
Differentiation of HD-NPC and shHD-NPC into astrocytes. (A) Astrocytes differentiated from HD-NPC expressing canonical astrocyte-specific markers, GFAP, ALDH1L1, and S100β. Staining with mEM48 showed nuclear and cytoplasmic aggregates of mHTT. (B) Astrocytes differentiated from shHD-NPC expressing canonical astrocyte-specific markers: GFAP, ALDH1L1, and S100β. Staining with mEM48 showed a lower number of cells that are positive for mEM48. (C) Quantification of ICC results showing all three cell lines expressed high percentage of cells with astrocyte specific markers ALDH1L1, GFAP, and S100β. HD cells showed significantly higher percentage of cells with PAX6-positive cells (17.6% vs. 2.20% WT and 9.8% shHD, P < 0.0001). shHD cells showed significantly higher percentage of cells with MAP2-positive cells (8.77% vs. 2.02% WT and 3.42%, P < 0.0001). In HD, mEM48 positive cell counts of HD and shHD astrocytes showing the significant reduced number of mEM48 positive cells in shHD astrocyte population (P < 0.0001, 23.9% and 2.56% respectively). For quantifications, minimum of 5 images and maximum of 24 images were counted in cellSens V2.1 (Olympus) with cell numbers ranging from 181 to 489 for each marker. A total of 2,167 cells of WT, 1,517 cells of HD, 1,727 cells of shHTT cells were counted.

Monkey NPCs were *in vitro* differentiated into astrocytes using a step-wise protocol (Fig 1A). Differentiated astrocytes displayed star-like appearance with extensive processes originating from the soma (Fig 1B). Several molecular hallmarks were chosen to confirm the genetic and functional identity of astrocytes. Astrocytes expressed canonical astrocyte-specific markers, glial fibrillary acidic protein (GFAP), aldehyde dehydrogenase 1 family member L1 (ALDH1L1), and S100 calcium-binding protein b (S100 β, but not NPC specific markers (paired box protein 6 (PAX6) and Musashi homolog 1 (MSI1)) and neural specific markers (microtubule associated protein 2 (MAP2)) (Fig 1C). The expression of cell-stage specific markers also showed the progressive changes in the protein expression (S1 Fig). The homogeneity of astrocytes was confirmed by fluorescence-activated cell sorting (FACS) analysis (Fig 1D) with over 96% positive for GFAP (96.7%, Fig 1D). After successful differentiation of WT-NPC, HD-NPC and shHD-NPC were differentiated into astrocytes using the same protocol (Fig 2A and 2B). HD and shHD astrocytes expressed canonical astrocyte-specific markers, ALDH1L1, GFAP, and S100β, while the expression of NPC specific marker (PAX6) and neural-specific marker (MAP2) were not observed, which correspond with real-time quantitative PCR data shown in next section (Fig 2A and 2B). Additionally, quantification of ICC data showed all three cell-lines showed high percentage of cells positive for astrocyte specific markers, while only few cell populations showed positive for either neuronal specific (MAP2) or NPC specific (PAX6) markers (Fig 2C). Immunostaining with mEM48, an antibody specific for expanded glutamine repeats of mHTT [22], showed nuclear inclusion of mHTT in HD astrocytes (Fig 2A) while a significantly lower number of mEM48 positive astrocytes was observed in shHD astrocytes (*P* < 0.0001) (Fig 2B and 2C).

### Gene expression of astrocytes derived from NPCs

**Fig 3:**
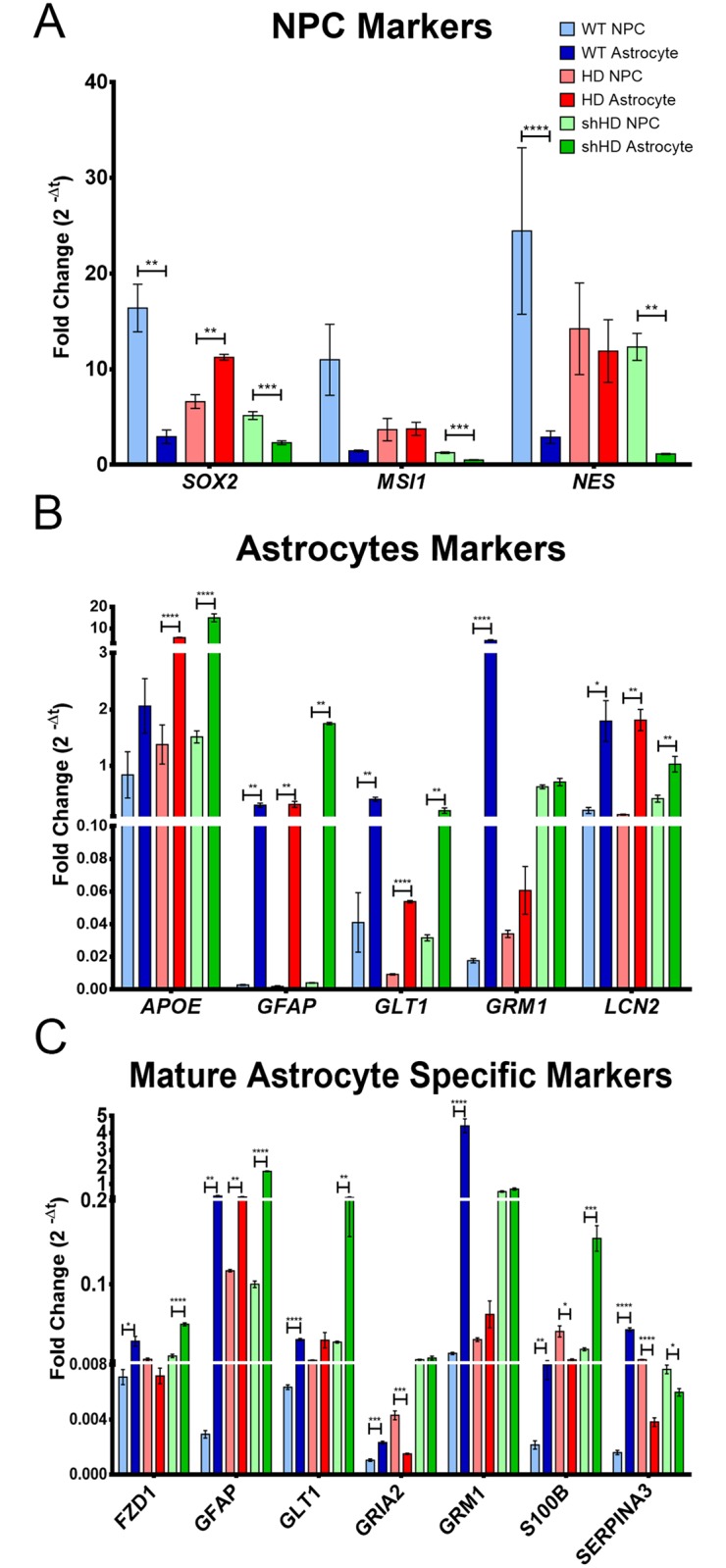
Gene expression profile of before and after astrocyte differentiation (day 30). (A) Before and after gene expression of NPC specific markers: *SOX2*, *MSI1*, and *NES*. WT and shHD showed a significant decrease in *SOX2* and *NES* expression. shHD showed a significant decrease in *MSI1* expression. (B) Before and after gene expression of astrocyte-specific markers: *APOE*, *GFAP*, *GLT1*, *GRM1*, and *LCN2*. All three cell lines showed a significant increase in *GFAP*, *GLT1*, and *LNC2* expression. HD and shHD showed a significant increase in *APOE* expression while only WT showed a significant increase in *GRM1* expression. (C) Mature astrocyte-specific marker expressions before and after the differentiation. All three cell lines showed significant increase in *GFAP* expression (WT *P* = 0.002, HD *P* = 0.003, shHD *P* < 0.0001), while only WT and shHD showed significant increase in *FZD1* (*P* = 0.012, *P* < 0.0001) and *S100β* (*P* = 0.006, *P* 0.001). HD showed significant decrease in *GRIA2* (*P* = 0.001), *S100β* (*P* = 0.008), and *SERPINA3* (*P* < 0.0001) expression. shHD showed significant decrease in *SERPINA3* (*P* = 0.016). At least two biological replicates and three technical replicates were analyzed for this study. Statistical significance was determined by ANOVA (asterisks denote following * *P* ≤ 0.05, ** *P* ≤ 0.01, *** *P* ≤ 0.001, and **** *P* ≤ 0.0001).

To further determine the identity of astrocytes derived from NPCs, the expression of a selected panel of cell type-specific genes during a 30 days astrocyte differentiation protocol (day 0,10, 20 and 30) were analyzed by RT-qPCR (S2 Fig) [19,29,30]. WT and shHD NPC derived astrocytes showed significant decrease in the expression of *SRY-related HMG-box 2* (*SOX2)* (*P* = 0.035 and *P* = 0.003, respectively) and *nestin* (*NES)* (*P* < 0.0001 and *P* = 0.001, respectively), NPC specific markers (Fig 3A) at the end of differentiation (day 30). shHD astrocytes also showed a significant decrease in *MSI1* expression at the end of differentiation (*P* = 0.001) (Fig 3A). However, HD astrocytes retained high expression of *SOX2* (*P* = 0.027) with no significant decrease in *MSI1* and *NES* expression (*P* = 0.961 and *P* = 0.727, respectively) (Fig 3A) at the end of differentiation. When astrocyte-specific gene expressions were compared at the end of differentiation (day 30), all three cell lines showed a significant increased expression of *GFAP*, *glutamate transporter 1* (*GLT1*), and *lipocalin 2* (*LCN2*) (Fig 3B). HD and shHD also showed significant increase in *apolipoprotein E* (*APOE*) expression (*P* = 0.0001 and *P* = 0.0001, respectively), while only WT showed significant increase in *glutamate metabotropic receptor 1* (*GRM1*) expression (*P* = 0.0001) (Fig 3B). All three cell lines did not express neuronal cell type-specific genes during and after differentiation (*MAP2*, *tubulin Beta 3 Class* III (*TUBB3*), *tyrosine hydroxylase* (*TH*), *glutamate decarboxylase 1* (*GAD*), *C-X-C motif chemokine receptor 1* (*CXCR1*), and f*orkhead box O4* (*FOXO4*) (S2 Fig).

To further evaluate the identity of the differentiated cells, we evaluated mature astrocyte gene expression before and after differentiation (Fig 3C). The markers were selected based on the literature[29,30,31] given the emphasis on functionality (glutamate uptake, trafficking, maintenance, and astrogliosis) of the astrocyte rather than structure. All three cell lines showed significant increase in *GFAP* expression at the end of differentiation (WT *P* = 0.002, HD *P* = 0.003, shHD *P* < 0.0001). However, only WT showed a significant increase in all six mature astrocyte markers (Fig 3C). shHD astrocytes showed a significant increase in *frizzled class receptor 1* (*FZD1*), *GFAP*, and *s100 calcium binding protein B* (*S100β*) but with a significant decrease in one of the markers, *serpin family A member 3* (*SERPINA3*) (Fig 3C). HD astrocytes did not have significant increase in the expression of the aforementioned markers except *GFAP* while a significant decrease in *glutamate ionotropic receptor AMPA type subunit 2* (*GRIA2*), *S100β*, and *SERPINA3* was observed (Fig 3C).

### Amelioration of HD cellular phenotypes by expressing shHD

**Fig 4:**
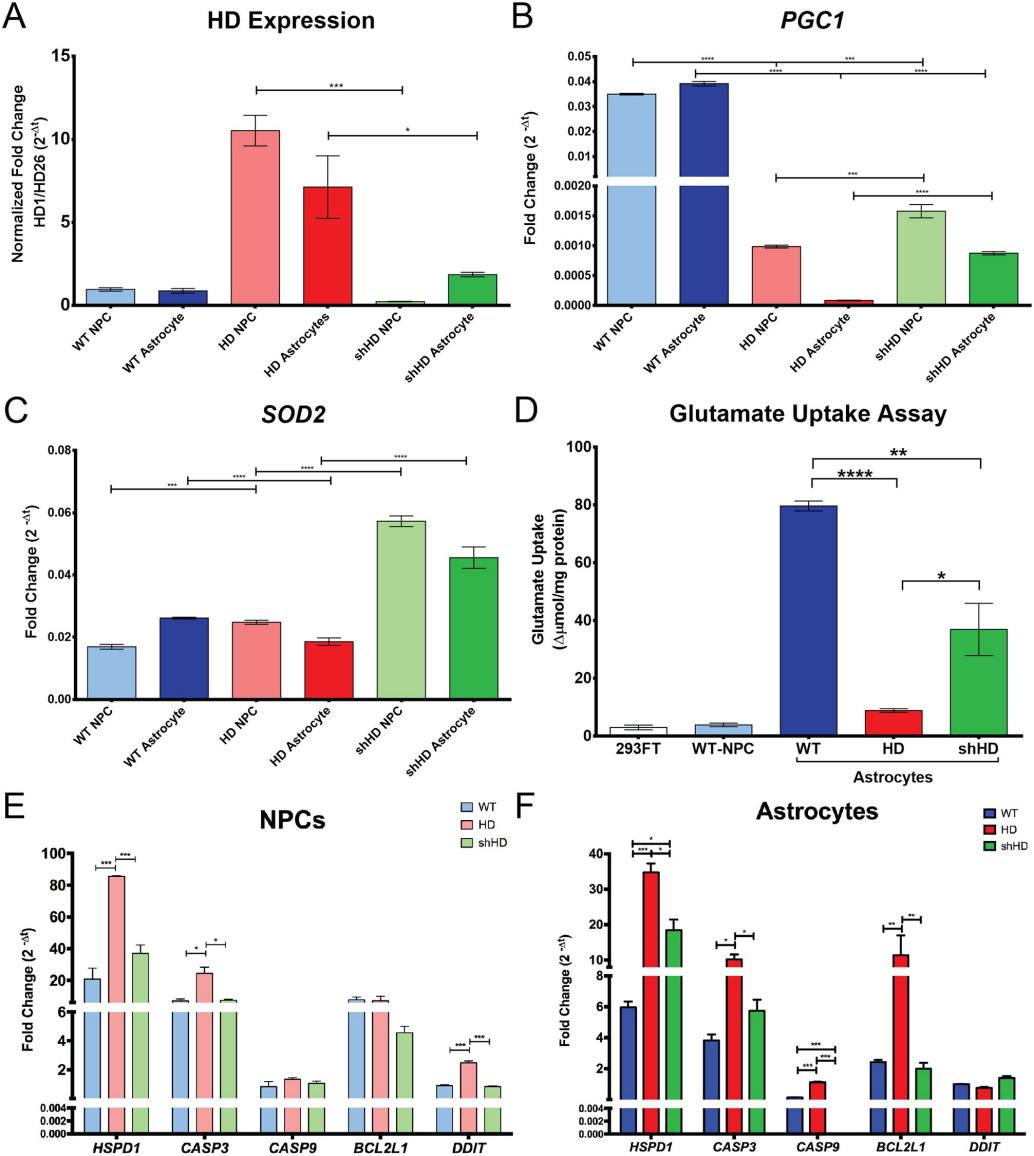
Expression of shHD showed a reversal of HD phenotype. (A) Expression of *shHD* in HD cells showed a reduction in *HTT* expression in astrocytes (*P* < 0.033). (B) Expression of *shHD* in HD cells induced expression of *PGC1* in astrocytes (*P* < 0.0001). (C) Expression of *shHD* in HD cells induced the expression of *SOD2* in astrocytes (*P* < 0.0001). (D) Glutamate uptake assay on differentiated astrocytes. Compared to WT both HD and shHD astrocytes showed significantly reduced the change in glutamate concentration in the supernatant (*P* < 0.0001 and *P* = 0.0097 respectively). However, shHD showed a significantly higher change in glutamate concentration in the supernatant (*P* = 0.036). Three biological replicates with three technical replicates were analyzed. (E) Expression of apoptosis associated genes in NPC showed significantly higher expression of *HSPD1* (*P* < 0.0001), *CASP3* (P ≤ 0.05), and *DDIT* (*P* < 0.0001) in HD NPC compared to both WT and shHD NPCs while no significant differences were found for *CASP9* and *BCL2L1* expression among all three cell lines. No expression differences between WT NPC and shHD NPC were observed in all markers. (F) Expression of apoptosis associated genes after the differentiation showed HD astrocytes expressed significantly higher levels of *HSPD1*, *CASP3*, *CASP9* and *BCL2L1* compared to both WT and shHD astrocytes. shHD astrocytes showed significantly higher expression of only *HSPD1* compared to WT astrocytes (P ≤ 0.05). All RT-qPCR data were analyzed with at least two biological replicates with three technical replicates. Statistical significance was determined by ANOVA and paired multiple *t*-test (asterisks denote following * *P* ≤ 0.05, ** *P* ≤ 0.01, *** *P* ≤ 0.001, and **** *P* ≤ 0.0001).

HD cells were also more susceptible to oxidative stress with less antioxidants and mitochondrial enzymes[32,33,34,35,36,37]. Dysregulated expression of peroxisome proliferator-activated receptor γ (PPARγ) co-activator 1α (*PGC1*), a regulator of mitochondrial biogenesis and oxidative stress, and superoxide dismutase 2 (*SOD2*), a mitochondrial enzyme that catalyzes superoxide radicals into hydrogen peroxide, have been reported in HD [38,39,40]. We have previously reported that expressing *shHD* in HD cells suppressed *mHTT* expression in NPC and neurons [19]. Similary, the expression of *shHD* suppressed the expression of *mHTT* in astrocytes (Fig 4A, *P* = 0.033). We also examined the expression of *mHTT*, *PGC1*, and *SOD2* in differentiated astrocytes (Fig 4A, 4B and 4C). Compared to WT astrocytes, HD astrocytes showed significantly lower expression of *PGC1* and *SOD1* (P < 0.0001 for both) (Fig 4B and 4C). Although shHD astrocytes showed significantly lower expression of *PGC1* compared to WT astrocytes (*P* < 0.0001) (Fig 4B), shHD astrocytes had significantly higher expression of *PGC1* and *SOD2* compared to HD astrocytes (*P* < 0.0001 for both) (Fig 4B and 4C). Since neuronal cell death is main pathogenesis of HD, we analyzed expressions of genes that are involved in apoptosis during differentiation process (Fig 4E and 4F, S3 Fig). HD cells showed significant increase expression of *HSPD1*, *CASP2* and *DDIT* compared to both WT and shHD in NPC stage (P < 0.0001, P ≤ 0.05, and P < 0.0001 respectively) (Fig 4E). After differentiation, HD astrocytes showed significantly higher expression of *HSPD1*, *CASP3*, *CASP9*, and *BCL2L1* compared to both WT and shHD (Fig 4F). Although shHD did not show any statistically significant different expression of apoptosis related gene at NPC stage compared to WT, shHD astrocytes showed significantly higher expression of HSPD1 (P ≤ 0.05) and significantly lower expression of CASP9 (P < 0.0001) (Fig 4F).

### Functionality of astrocytes derived from neural progenitor cells

Astrocyte functions were evaluated by glutamate uptake assay and electrophysiology. Since glutamate clearance is one of the primary functions of astrocytes, we measured glutamate uptake capability of differentiated astrocytes. As shown in Fig 4, WT astrocytes showed the highest uptake of glutamate in culture (79.63 Δμmol/mg protein). Compared to WT, both HD and shHD astrocytes showed much lower glutamate uptake (*P* < 0.0001 and *P* = 0.0097) (Fig 4D), while shHD astrocytes has much higher glutamate uptake capability compared to HD astrocytes (*P* = 0.036) (Fig 4D).

**Fig 5:**
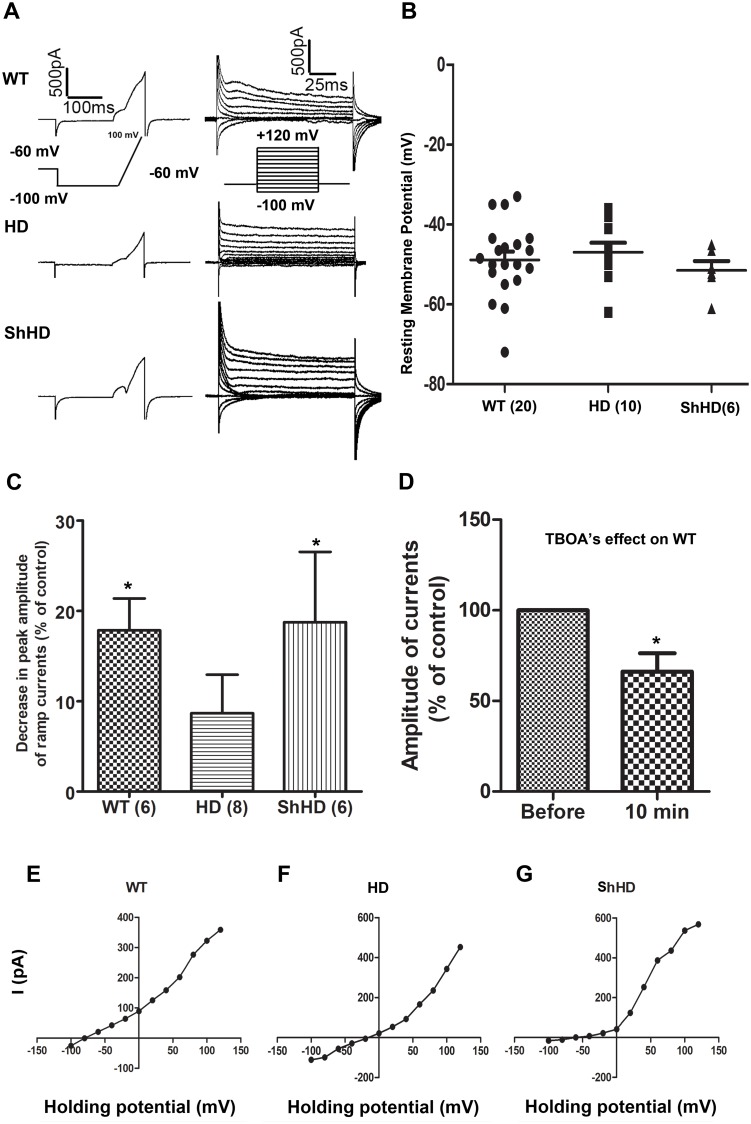
Functional characterization of differentiated astrocytes. (A) Representative traces of ionic currents evoked by ramp protocol (left) and voltage-step protocol (right) in cultured WT, HD, and shHD astrocytes. The holding potentials for all the cells were -60mV. (B) Plots of resting membrane potential values for the three types of cells recorded before the current membrane activation. There is no statistical significance among three groups. (C) Percentage of ramp current decrease measured at +100 mV by application of 4-AP (500 uM) in the superfusion medium during recordings on all three types of astrocytes. (D) Significant decreases in the amplitude of peak ramp currents were observed in WT astrocytes following 20 min application of TBOA at the concentration of 500 uM (n = 6). (E, F, G) I/V plots show that all three types of astrocytes share the similarity with variably rectifying astrocyte reported in the literature. However, the reversal potential of (F) HD astrocytes was significantly shifted compared with those in both (E) WT and (G) shHD cells. At least four biological replicates were included for each cell line, and n represents each individual reading.

Electrophysiological properties were examined by whole-cell patch-clamp recordings. We compared their ability to generate K^+^ currents upon voltage steps and ramp stimulations stimulation (Fig 5A). Ramp currents (Fig 5A, left panel) were induced in all three types of astrocytes. Moreover, upon application of voltage-step protocol, typical outward K^+^ currents were evoked in all three types of astrocytes (Fig 5A, right panel, WT—n = 20, HD—n = 10, and shHD—n = 6). All three astrocytes showed negative resting membrane potential (RMP) (WT: -48.29 ± 2.18 mV, n = 20; HD: -46.96 ± 1.69 mV, n = 10; ShHD: -51.47 ± 1.27 mV, n = 6) with no statistically significant differences among groups (Fig 5B). Since out ward currents induced by voltage ramp protocol strongly suggests K^+^ conductance, current strongly suggests K^+^ upon voltage ramp and steps stimulations, all three cell lines were treated with K^+^ channel blocker 4-aminopyridine (4AP), a non-selective voltage dependent K^+^ channel blocker to dissect the currents (Fig 5C). WT and shHD showed significantly decreased amplitude in ramp currents after 20 min 4AP treatment at a concentration of 500 uM (WT: 17.84 ± 3.84%, n = 6; HD: 8.66 ± 4.03%, n = 8; ShHD: 18.76 ± 8.51%, n = 6, *P < 0.05) (Fig 5C). Although HD showed a slight decrease in ramp currents after 4AP treatment, it was not statistically significant (Fig 5C). We also examined the involvement of glutamate transporter in this ramp current by blocking glutamate uptake with DL-threo-benzyloxyaspartic acid (TBOA). WT astrocytes showed significant decrease in the peak amplitude of ramp currents after 10 min of TBOA treatment at a concentration of 200 uM (66.15 ± 11.13%, n = 6, *P < 0.05). Current-to-voltage (I/V) plots showed that all three types of astrocytes share similarity with variably rectifying astrocyte reported in the literature[41,42] (Fig 5E, 5F and 5G). However, the reversal potential of HD astrocytes was significantly shifted to the right compared with those in both WT and shHD cells (Fig 5F). I/V plot of shHD showed a shift in the reversal potential of evoked currents to the positive direction, which indicates there may be a marked change on the activity of the K^+^ channels in those astrocytes (Fig 5G).

## Discussion

Here we present an efficient method to derive functional astrocytes from monkey NPC. This protocol generates homogeneous astrocytes from NPC *in vitro*. The astrocytes expressed canonical astrocyte specific markers (Fig 1) and were functionally competent. We also differentiated HD astrocytes using the same protocol and assessed disease-specific phenotypes. Generating disease specific cell population is of interest in discovering novel treatment targets and developing genetic and pharmacological therapies. The differentiation method used in this study is relatively short compared to other protocols (~ 6 months) [43,44,45] and compatible with commercial astrocyte differentiation products (STEMdiff^™^ by STEMCELL^™^—30 days). The ease and short-term differentiation protocol used in this study can generate homogeneous population of astrocytes with functional characteristics are important for treatment development.

Modeling disease *in vitro* has been explored in recent years in conjunction with the development of iPSC technology and directed cell-reprogramming technology. Faithful recapitulation of disease phenotype is particularly important in terms of therapy evaluation and drug discovery. HD astrocytes generated in this study recapitulate many known HD cellular phenotypes such as mHTT aggregates [46], suppressed expression of stress response genes (*PGC1* and *SOD2*) [38], inefficient glutamate clearance [47,48], and aberrant electrophysiology [16]. Increased intranuclear inclusions and accumulation of cytoplasmic mHTT aggregates are the hallmarks of HD pathology [46]. Although the impact of mHTT in astrocytes remains to be elucidated, mHTT aggregates in glial cells in HD have been reported [48,49]. Many HD astrocytes formed mHTT aggregates and nuclear inclusion as shown by mEM48 staining (Fig 2). Oxidative stress and mitochondrial dysfunction have shown in neurodegenerative diseases [40]. Expression of *PGC1* and *SOD2* were both suppressed in R6/2 HD mice, which involved in mitochondrial function and resistance to oxidative stress, respectively [38]. *PGC1* is required for the induction of *SOD2*, which is important in detoxifying reactive oxygen species (ROS) [39]. Both PGC1^-/-^ and SOD2^+/-^ mouse models showed neurodegenerations [39,40]. HD astrocytes showed significantly reduced expression of both *PGC1* and *SOD2*, which are consistently with prior reports (Fig 4). Mitochondrial dysfunction is also associated with neurodegenerative disease and has been suggested as therapeutic target to ameliorate neurodegeneration [50,51]. Glutamate excitotoxicity is an important pathogenic mechanism in HD [47]. One of the main functions of astrocyte is the clearance of glutamate in neural synapsis and prevent excitotoxicity. To determine the integrity and functions of astrocytes, we examined the expression of genes that are responsible or associated with glutamate uptake which include *GLT1*, *GRM1*, glutamate metabotropic receptor 5 (*GRM5*), and *GRIA2*, and glutamate uptake capacity. As expected, HD astrocytes showed lower expression of *GLT1*, *GRM1*, *GRM5*, and *GRIA2* (Fig 3B and 3C). Moreover, glutamate uptake assay showed significantly lower glutamate uptake ability in HD astrocytes (Fig 4D). Corticostriatal dysfunction and reduced activity of *GLT1* (*EAAT2* or *slc1a2*) is well documented in mouse HD brain, which directly correlate with glutamate uptake by astrocytes [47,48,52,53]. Moreover, the expression of *GLT1* is regulated by *SP1* [54], which binds with mHTT [54]. Ionotropic and metabotropic glutamate receptor genes (*GRIN*, *GRIA*, and *GRM*) are regulated by similar epigenetic mechansim at H3-(methyl)-lysine 4 site [55] as well. Neuronal cell death is one of the hallmarks of Huntington’s disease, and apoptosis has long been suspected to play a role in the process. Activation of caspase 3 (*CASP3*) and caspase 9 (*CASP9*) were observed in HD [56,57]. B-cell lymphoma 2 (BCL-2) proteins regulate apoptotic mitochondrial pathway and has shown to aberrantly expressed and localized in *in vivo* and *in vitro* models of HD[58]. Also, heat shock 60 kDa protein 1 (*HSPD1*) is up-stream effector of *CASP3* and has been reported to show elevated expression in grade 3 and grade 4 HD caudate nucleus in HD patients[37]. As a late apoptotic marker, DNA-damage-inducible transcript (DDIT) was included in this study. As we expected, HD cells showed higher expression of apoptosis response genes such as *HSPD1*, *CASP3*, and *DDIT* at NPC stage compared to both WT and shHD cell lines (Fig 4E). All three cell lines generally showed decrease in apoptosis related gene expression during differentiation process (S3 Fig). After differentiation, HD astrocytes showed significantly higher expressions of *HSPD1*, *CASP3*, *CASP9*, and *BCL2L1* compared to WT and shHD astrocytes (Fig 4F). Therefore, HD astrocytes differentiated from monkey iPSC recapitulated cellular and molecular biological hallmarks of Huntington’s disease.

*In vitro* electrophysiology showed loss of voltage dependent K^+^ conductance in HD astrocytes compared with both WT and shHD astrocyte (Fig 5A). HD astrocytes treated with 4AP, a potassium channel blocker, did not respond as well as WT and shHD astrocytes (Fig 5C and 5D), which suggests reduced K^+^ channel function. Recently, decreased Kir4.1 K^+^ ion channel function was observed in HD mouse models [16]. Taken together, our data support recapitulation of many HD astrocyte phenotypes and our model hold great promise for studying HD pathogenesis.

As a potential platform for developing new therapies, we investigated if RNAi can reduce HTT expression and ameliorate HD cellular phenotypes. RNAi have shown promising outcomes in number of studies with two ongoing clinical trials in HD [19,59,60,61,62,63]. Our prior report demonstrated that overexpression of shHD in HD neurons ameliorates HD phenotypes such as reduced oligomeric aggregates, reduced intranuclear inclusions, and improved cell survival under oxidative stress [19]. Consistently, cellular phenotypes in HD astrocytes were ameliorated by the expression of shHD. shHD astrocytes showed reduced mHTT aggregates and intranuclear inclusions (Fig 2), restored gene expressions (Figs 3 and 4), improved glutamate uptake ability (Fig 4D), and improved electrophysiological properties of inwardly rectifying K^+^ channel (Fig 5). Unlike gene editing technologies, such as CRISPR-Cas9, TALEN, and ZFN, RNAi is inherently limited to partial suppression of the target gene. In our study, *mHTT* was partially suppressed (Figs 2 and 4) that some of the HD phenotypes were not completely restored; *SERPINA3*, *PGC1*, and *GRM1* gene expressions remain low compared to WT astrocytes, and *HSPD1* expression remained high compared to WT astrocytes (Figs 3 and 4), similar effect was also observed in glutamate uptake (Fig 4D) and I/V plot showed shifted reversal potential of evoked currents (Fig 5G) compared to WT astrocytes.

HTT plays important roles in neural development and neurological functions [1]. Thus, preserving HTT expression and function is important to normal cell functions. A recent study showed conditional knockout of HTT in adult neuron showed no deleterious impact in mouse model while early deletion of HTT resulted in early death [64]. Expressing mHTT in only astrocytes showed similar neurological symptoms as HD [54]. It is unknown whether complete ablation of HTT in astrocyte will be deleterious. Therefore, RNAi might provide a better approach as of now. With more precise gene editing tools and personalized medicine becomes available, allele specific gene editing/silencing and gene correction through homologous recombination might provide better methods to treat HD. Here we demonstrated an *in vitro* astrocyte platform to evaluate efficacy of RNAi on HD astrocyte. This platform could be useful for developing new treatment and assessing impact on astrocytes.

Astrocytes are heterogenic and a dynamic group of neural cells with different morphologies and functions [29,65]. Specifically, astrocytes can be classified as reactive and non-reactive astrocytes [29,65]. Reactive astrocytes are classified by elevated expression of GFAP, nestin, vimentin, aquaporins, proteoglycans, and GRM5 [29,66]. Non-reactive astrocytes express low GFAP and high GLT1 and GLAST [67]. Based on morphology, gene expression profile, low glutamate uptake ability, and electrophysiology, HD astrocytes resemble reactive astrocytes, while WT and shHD astrocytes resemble non-reactive astrocytes. In a follow-up study, we have examined transcriptomic profile of *in vitro* differentiated WT and HD astrocytes that HD astrocytes exhibited upregulation of A1-specific genes while down regulation of A2-specific genes were observed (unpublished data). A1-specific astrocytes are more abundant in neurodegenerative diseases including HD, activated by microglia, and are neurotoxic [68]. WT and shHD astrocytes have increased expression of *GLT1*, *GRM1*, *GRM5*, and *FZD1* (Fig 3B and 3C) compared to that of HD astrocytes. HD astrocytes maintained high expression levels of *NES*, *MSI1* and *SOX2* (Fig 3A). However, gene expression pattern throughout astrocyte differentiation showed elevated expression of *GLT1* and *GRM1*, and reduced expression of *SOX2*, *NES*, and *MSI1* in early stage of differentiation in HD astrocytes (S2 Fig). In fact, HD astrocyte differentiation has the lowest yield compared to WT and shHD groups which might due to reduced competence of HD NPC in astrocyte differentiation (data not shown).

Mutant HTT affects a broad spectrum of astrocyte functions. Examining pathological pathways, such as apoptosis cascades with additional time-course studies on genomic, proteomic and epigenetic assessment will further elucidate the pathogenic mechanisms in HD astrocytes. Although this study is primarily focused on apoptosis pathway as the major pathogenic response to mHTT in astrocytes, other pathways are remained to be investigated in future studies. Since striatal medium spiny neurons (MSNs) are the main target of the mHTT, many pathological pathways have been suggested in MSNs pathogenesis which include the suppression of brain-derived neurotrophic factor (BDNF) [69], excitotoxity arising from glutamatergic cortical projections [70], and and interaction with Rhes protein [71]. In fact, many other pathogenic mechanisms such as disruption in autophagy [72], aberrant ubiquitin-proteasome activity [73], protein aggregations [46,74], aberrant transcription [75], impaired TrkB postsynaptic signaling [76], impaired vesicular transport [77], impaired calcium trafficking [78], aberrant metabolism [79], mitochondrial dysfunction [80], heightened inflammatorily response [81], and inhibition of TEAD/TAP [82] are all been reported in HD. Thus a thorough investigation using an *in vitro* model system such as our *in vitro* NHP astrocyte model will help delineate pathogenesis on HD during neurodevelopment.

A recent study showed astrocytes differentiated from human iPSC derived NPCs resembles gene expression profile of quiescent astrocytes [83], which is consistent with our WT and shHD astrocytes that exhibited gene expression profile similar to quiescent astrocytes. However, HD astrocytes were more resemblance to reactive astrocytes. Whether this is due to *in vitro* differentiation process or diseases specific phenotype remains to be investigated.

Astrocytes have been used in cell replacement therapy for treating various neurological diseases such as amyotrophic lateral sclerosis (ALS), Alzheimer’s disease, Parkinson’s disease, and Huntington’s disease [84,85]. Co-transplanting astrocytes with NPC has also shown to improve therapeutic outcome in Parkinson’s disease mouse model [86]. While these reports are encouraging for future development of cell replacement therapy, more vigorous assessment with relevant clinical measures are important prior to clinical translation. Cognitive and emotional impact are important clinical measures in neurodegenerative diseases such as HD which are often difficult to assess in rodent models. Recent reports on longitudinal assessment of HD monkeys have further suggested their potential as preclinical animal models because of similar disease progression and accessible clinical measurements that readily for clinical translation [87,88,89,90,91]. Thus our NHP HD astrocyte model and HD monkey model could provide a unique platform for preclinical assessment of cell replacement therapy in the future. In summary, our *in vitro* astrocyte model provides a unique cell source for the development of cell replacement therapeutic approach and potenatil for preclinical assessment in HD monkeys. Here, we have demonstrated the modeling of HD in *in vitro* derived monkey astrocytes and evaluated therapeutic efficacy RNAi as a proof principle. Our method can be utilized in other central nerve system (CNS) diseases in studying of the role of astrocytes in disease pathogenesis, developing therapies, and assessment of novel treatment.

## Supporting information

S1 Fig Expression of cell-stage specific markers during the differentiation process. (TIF)

S2 Fig Gene expression analysis during astrocyte differentiation. (A-C) WT, HD, and shHD, respectively, gene expression changes during the astrocyte differentiation. (All samples were analyzed at least in triplicates. Statistical significance was determined by ANOVA with Bonferonni test post- (asterisks denote following * P ≤ 0.05, ** P ≤ 0.01, *** P ≤ 0.001, and **** P ≤ 0.0001).
(TIF)

S3 Fig Expression of apoptosis associated markers during the differentiation process. (TIFF)
