# Supplementary figures and images for: Amelioration of Huntington’s disease phenotype in astrocytes derived from iPSC-derived neural progenitor cells of Huntington’s disease monkeys
Source: PLoS One. 2019 Mar 21;14(3):e0214156. doi: 10.1371/journal.pone.0214156 (PMC6428250; doi:10.1371/journal.pone.0214156)

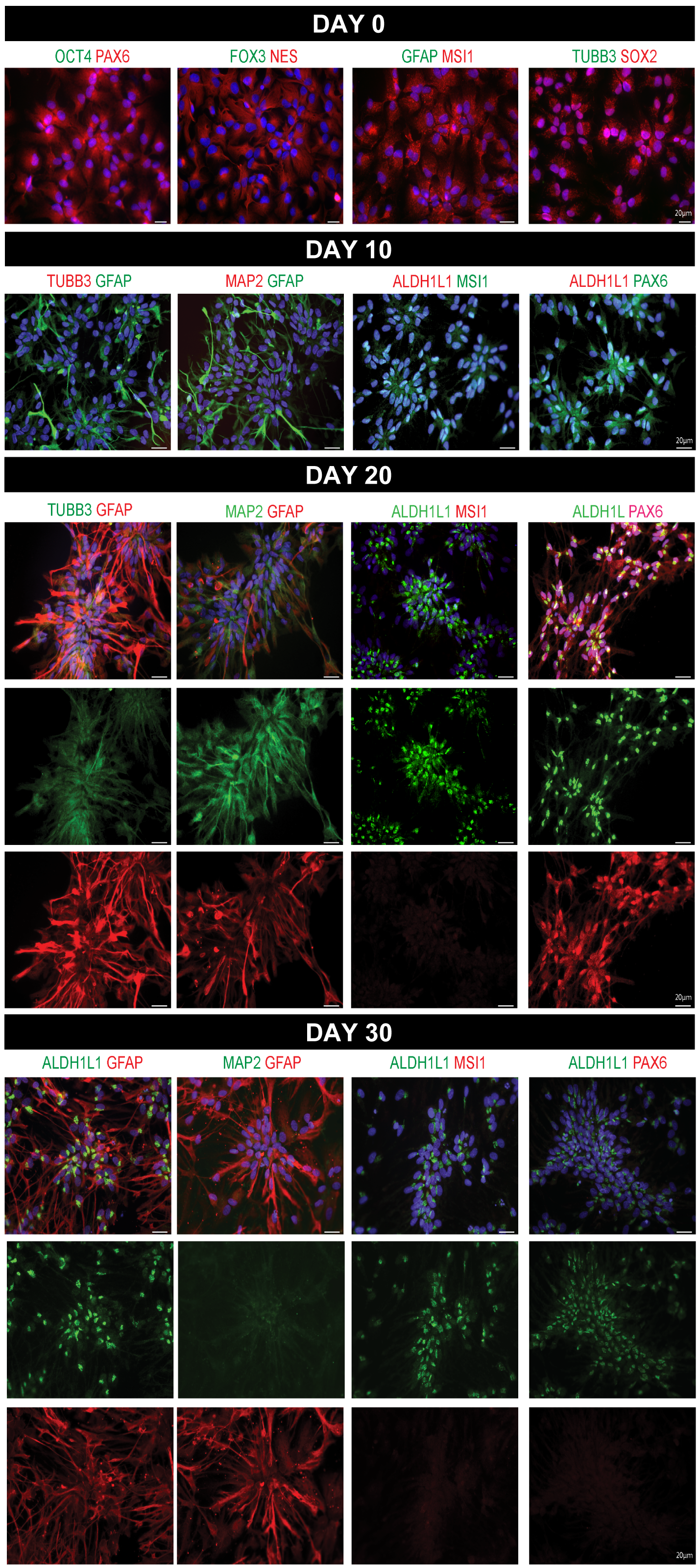

Supplement: S1 Fig — (TIF) [file pone.0214156.s001.tif]

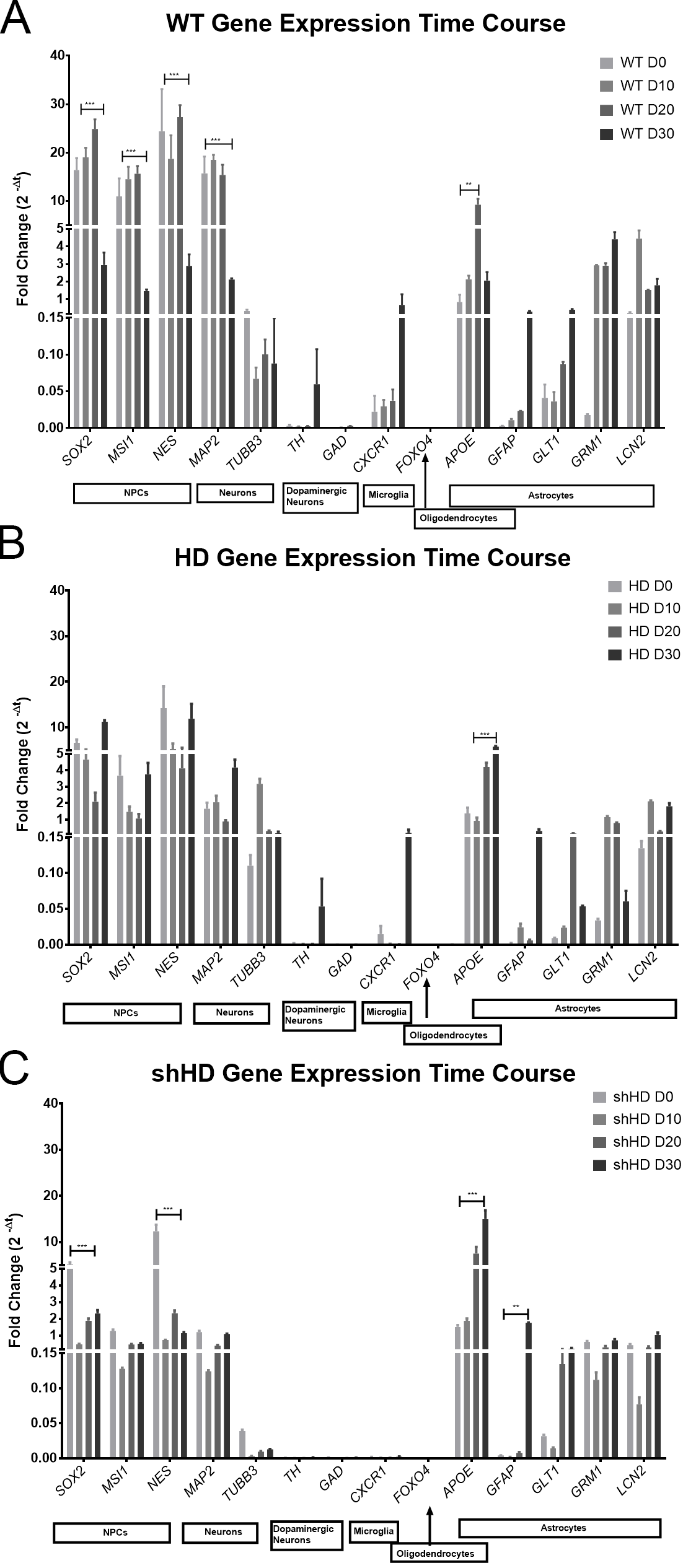

Supplement: S2 Fig — (A-C) WT, HD, and shHD, respectively, gene expression changes during the astrocyte differentiation. (All samples were analyzed at least in triplicates. Statistical significance was determined by ANOVA with Bonferonni test post- (asterisks denote following * P ≤ 0.05, ** P ≤ 0.01, *** P ≤ 0.001, and **** P ≤ 0.0001). (TIF) [file pone.0214156.s002.tif]

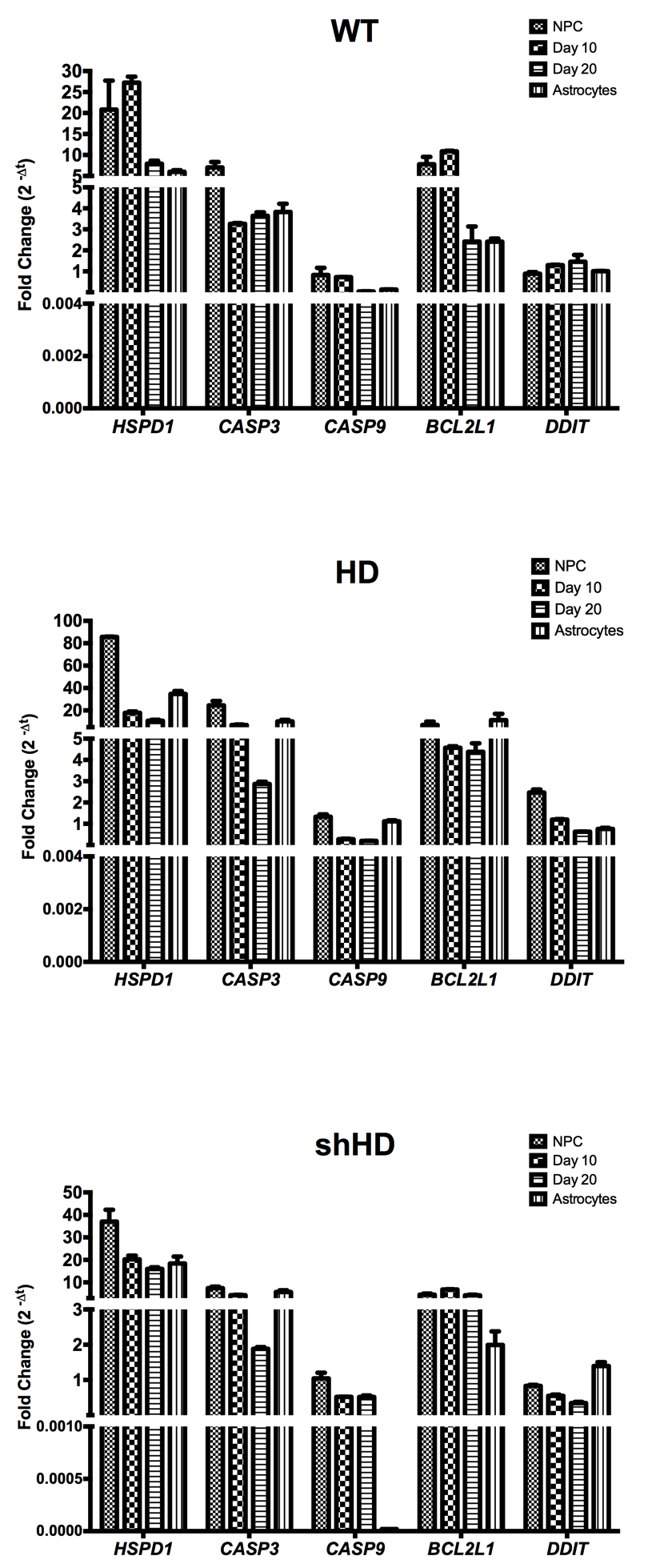

Supplement: S3 Fig — (TIFF) [file pone.0214156.s003.tiff]
